# An Iterative Image Registration Algorithm by Optimizing Similarity Measurement

**DOI:** 10.6028/jres.115.001

**Published:** 2010-02-01

**Authors:** Wei Chu, Li Ma, John Song, Theodore Vorburger

**Affiliations:** National Institute of Standards and Technology, Gaithersburg, MD 20899

**Keywords:** image registration, Newton-Raphson iteration, rigid body transformation, similarity measurement, standard casing

## Abstract

A new registration algorithm based on Newton-Raphson iteration is proposed to align images with rigid body transformation. A set of transformation parameters consisting of translation in *x* and *y* and rotation angle around *z* is calculated by optimizing a specified similarity metric using the Newton-Raphson method. This algorithm has been tested by registering and correlating pairs of topography measurements of nominally identical NIST Standard Reference Material (SRM 2461) standard cartridge cases, and very good registration accuracy has been obtained.

## 1. Introduction

Image registration is a process of determining the point-by-point correspondence between two or more images taken of a scene at different times, by different sensors, or from different points of view. The images are aligned with one another so that differences between them can be detected. The method has applications in many areas such as remote sensing, medical imaging, computer vision, image similarity measurement, etc. Based on different criteria, registration methods and applications can be classified differently. Over the years, a broad range of techniques has been developed for registration of various types of data and images [1, 2].

The parameters that make up the registration transformation can either be computed directly, if possible, or be determined by finding an optimum of some function defined on the parameter space. In the latter case, the transformation parameters can be incorporated into a standard mathematical function. This function attempts to quantify the similarity between two images given a certain transformation [2]. The goal of image registration is to find the optimum parameters to make the function reach its extremum. Various optimization techniques can be used to accomplish this. As a well-known numerical analysis method for finding successively better approximations to the zeroes of a real-valued function, the Newton-Raphson method is one of the widely used optimization techniques [3]. With this method applied, Lucas and Kanade [4] presented a registration technique making use of the spatial intensity gradient of images, Vendroux and Knauus [5] optimized a digital image correlation (DIC) coefficient to calculate surface displacements and displacement gradients of a sample under deformation, and Cole-Rhodes et al. [6] calculated a mutual information metric, which may be especially useful for registration of multi-modality images (i.e., images taken by different imaging methods) [7].

In response to a request from the Bureau of Alcohol, Tobacco, Firearms, and Explosives (ATF) to develop physical standards for bullets and casings for use in the validation of image analysis systems for ballistics identification, the National Institute of Standards and Technology (NIST) has developed the Standard Reference Material (SRM 2460) standard bullets and 2461 standard cartridge cases [8]. The similarity between the master standard bullet or cartridge case and their replicas, or between different replicas needs to be determined. This calculation requires an explicit similarity metric. A parameter called the signature difference *D_s_* has been proposed by NIST to quantify the similarity for both 2D and 3D ballistics signatures [9]. Accordingly, an appropriate image registration method is needed to align two casing images before the similarity between them is calculated. For the practical requirement of determining the reproducibility of the topography signatures of bullets or cases, a new image registration method based on Newton-Raphson iteration is developed, and this is directly associated with the signature difference parameter. In this paper, we introduce the mathematical algorithm in Sec. 2, we define measures of similarity in Sec. 3, and we describe the experimental tests in Sec. 4.

## 2. The Mathematical Algorithm

In image processing and computer vision, rigid body transformation can be decomposed into a rotation and a translation. We assume that the topographic images are embedded in a common coordinate system. If the transformation is in a 2D plane, which is a common simplification in many optical image processing and measurement situations, the coordinate position (*x*, *y*) of any point inside the object being transformed will be relocated to $\left(\tilde{x},\tilde{y}\right)$ according to

(1) $$\left[\begin{array}{c} \tilde{x}\\ \tilde{y} \end{array}\right]=\left[\begin{array}{cc} \cos\theta & -\sin\theta \\ \sin\theta & \cos\theta \end{array}\right]\left[\begin{array}{c} x\\ y \end{array}\right]+\left[\begin{array}{c} T_{x}\\ T_{y} \end{array}\right]$$

or

(2) $$\{\begin{array}{c} \tilde{x}=T_{x}+x\cos\theta -y\sin\theta \\ \tilde{y}=T_{y}+x\sin\theta +y\cos\theta \end{array}.$$

We assume that *f* and *g*, which are called respectively the reference image and the comparison image in this paper, are images acquired before and after the object is physically transformed and that the transformation is that of a rigid body. Let **P** represent the array of all the parameters of the transformation. In the case of the 2D rigid body transformation as defined by Eq. (2), **P** is composed of three transformation parameters, *T_x_*, *T_y_*, and *θ*. For each transformation **P**, image *g* will be uniquely determined, and vice versa. When the reference image, *f*, and the comparison image, *g*, are given and the transformation array needs to be solved, a registration technique is required. The object of registration is to determine the rigid body transformation **P** that aligns the two acquired images, *f* and *g*, as well as possible.

Let *S_p_* represent any single point in the reference image *f* with the lateral coordinates (*x*, *y*) and $\bar{P}$ an estimation value of the real transformation **P**. For each estimation $\bar{P}$, *S_p_* has a *corresponding* point in the comparison image, which is denoted as ${\tilde{S}}_{p}$. The intensity values of *S_p_* and ${\tilde{S}}_{p}$ can be denoted as *f* (*S_p_*) and $g(S_{p},\bar{P})$, or equivalently as *f* (*x*, *y*) and $g(\tilde{x},\tilde{y})$. We use a parameter *C* to quantify how well the two images match. It is defined by the ratio of sum of squared intensity differences between the two images to the sum of squared intensities of the original image, which is taken as the reference. See Eq. (3).

(3) $$C=\frac{\sum_{S_{p}\in f,{\tilde{S}}_{p}\in g}{\left[f(S_{p})-g(S_{p},\bar{P})\right]}^{2}}{\sum_{S_{p}\in f,{\tilde{S}}_{p}\in g}f^{2}(S_{p})}.$$

*C* is a function of $\bar{P}$, the array of estimated transformation parameters. When the correct **P** that transforms the object is substituted into Eq. (3), *C* will reach its minimum. The minimum should be zero for the ideal condition that noise and error are not considered. Conversely, an estimation $\bar{P}$ that minimizes *C* is the correct one.

The pixels of both acquired reference and comparison images are at grid positions. However, the coordinate positions $\left(\tilde{x},\tilde{y}\right)$ of points in the comparison image mapped by $\bar{P}$ may not be at grid points. Therefore, in order to calculate the value of *C*, an interpolation process is required to define the function value *g* at non-grid positions. In order to obtain good piecewise continuity and smoothness of the interpolation function and avoid oscillation of the function, a bi-cubic spline interpolation [10] is applied using common methods [11]^1^ for each choice of the array $\bar{P}$.

Now the goal to find an estimation array $\bar{P}$ to minimize the function *C* is an optimization problem. To find the minimum of *C*, the gradient of *C* with respect to variation in the parameters of $\bar{P}$ must be zero. If *T_x_*, *T_y_*, and *θ* are denoted as *p_i_*(*i* = 1,2,3), we will get

(4) $$\begin{array}{l} \nabla C=\frac{-2}{\sum_{S_{p}\in f,{\tilde{S}}_{p}\in g}f^{2}(S_{p})}\\ {\left\{\sum_{S_{p}\in f,{\tilde{S}}_{p}\in g}\left[f(S_{p})-g(S_{p},\bar{P})\right]\frac{\partial g(S_{p},\bar{P})}{\partial p_{i}}\right\}}_{i=1,2,3}=0 \end{array}$$

where

(5) $$\begin{array}{l} \frac{\partial g(S_{p},\bar{P})}{\partial p_{1}}=\frac{\partial g(S_{p},\bar{P})}{\partial \tilde{x}}\cdot \frac{\partial \tilde{x}}{\partial p_{1}}\\ +\frac{\partial g(S_{p},\bar{P})}{\partial \tilde{y}}\cdot \frac{\partial \tilde{y}}{\partial p_{1}}=\frac{\partial g(S_{p},\bar{P})}{\partial \tilde{x}}\\ \;\frac{\partial g(S_{p},\bar{P})}{\partial p_{2}}=\frac{\partial g(S_{p},\bar{P})}{\partial \tilde{y}}\\ \frac{\partial g(S_{p},\bar{P})}{\partial p_{3}}=\frac{\partial g}{\partial \tilde{x}}\cdot [x\cdot \sin(-p_{3})-y\cdot \cos(p_{3})]\\ \;+\frac{\partial g}{\partial \tilde{y}}\cdot [x\cdot \cos(-p_{3})-y\cdot \sin(p_{3})]. \end{array}$$

The nonlinear Eq. (4) can be solved using the Newton-Raphson iteration method [3]. Then, we obtain

(6) $${\bar{P}}^{k+1}={\bar{P}}^{k}-\nabla C({\bar{P}}^{k})\cdot {\left[\nabla \nabla C({\bar{P}}^{k})\right]}^{-1}$$

where ${\bar{P}}^{k}$ is the *k*^th^ iteration value and ${\bar{P}}^{k+1}$ is the approximation value after the (*k* + 1) iteration. $\nabla \nabla C(\bar{P})$ is the Hessian matrix [12].

(7) $$\nabla \nabla C(\bar{P})={\left(\frac{\partial^{2}C}{\partial p_{i}\partial p_{j}}\right)}_{i,j=1,2,3;}$$

Based on Eq. (4), it can be expanded as

(8) $$\begin{array}{l} \nabla \nabla C(\bar{P})=\frac{-2}{\sum_{S_{p}\in f,{\tilde{S}}_{p}\in g}f^{2}(S_{p})}\\ \;\left\{\sum_{S_{p}\in f,{\tilde{S}}_{p}\in g}[f(S_{p})-g(S_{p},\bar{P})]\frac{\partial^{2}g(S_{p},\bar{P})}{\partial p_{i}\partial p_{j}}\right\}\\ +\frac{2}{\sum_{S_{p}\in f,{\tilde{S}}_{p}\in g}f^{2}(S_{p})}\\ \left\{\sum_{S_{p}\in f,{\tilde{S}}_{p}\in g}\frac{\partial g(S_{p},\bar{P})}{\partial p_{i}}\frac{\partial g(S_{p},\bar{P})}{\partial p_{j}}\right\} \end{array}$$

where *i*, *j* = 1,2,3.

If $\frac{\partial^{2}g(S_{p},\bar{P})}{\partial p_{i}\partial p_{j}}$ are denoted as *H_ij_*, they will be

(9) $$\begin{array}{l} H_{11}=\frac{\partial^{2}g(S_{p},\bar{P})}{\partial {\tilde{x}}^{2}};\;H_{12}=\frac{\partial^{2}g(S_{p},\bar{P})}{\partial \tilde{x}\partial \tilde{y}};\\ H_{21}=\frac{\partial^{2}g(S_{p},\bar{P})}{\partial \tilde{y}\partial \tilde{x}};\;H_{22}=\frac{\partial^{2}g(S_{p},\bar{P})}{\partial {\tilde{y}}^{2}}\\ H_{13}=H_{11}\cdot [x\cdot \sin(-p_{3})-y\cdot \cos(p_{3})]\\ \;+H_{12}\cdot [x\cdot \cos(p_{3})-y\cdot \sin(p_{3})]\\ H_{23}=H_{21}\cdot [x\cdot \sin(-p_{3})-y\cdot \cos(p_{3})]\\ \;+H_{22}\cdot [x\cdot \cos(p_{3})-y\cdot \sin(p_{3})]\\ H_{31}=H_{11}\cdot [x\cdot \sin(-p_{3})-y\cdot \cos(p_{3})]\\ \;+H_{21}\cdot [x\cdot \cos(p_{3})-y\cdot \sin(p_{3})]\\ H_{32}=H_{12}\cdot [x\cdot \sin(-p_{3})-y\cdot \cos(p_{3})]\\ \;+H_{22}\cdot [x\cdot \cos(p_{3})-y\cdot \sin(p_{3})]\\ H_{33}=\\ \;\{H_{11}\cdot [x\cdot \sin(-p_{3})-y\cdot \cos(p_{3})]\\ \;+H_{12}\cdot [x\cdot \cos(p_{3})-y\cdot \sin(p_{3})]\}\\ \;\cdot [x\cdot \sin(-p_{3})-y\cdot \cos(p_{3})]+\\ \;\cdot [x\cdot \cos(p_{3})-y\cdot \sin(p_{3})]+\\ \;\{H_{21}\cdot [x\cdot \sin(-p_{3})-y\cdot \cos(p_{3})]+\\ \;H_{22}\cdot [x\cdot \cos(p_{3})-y\cdot \sin(p_{3})]\}\\ \;\cdot [x\cdot \cos(p_{3})-y\cdot \sin(p_{3})]+\\ \;\frac{\partial g(S_{p},\bar{P})}{\partial \tilde{x}}\cdot [-x\cdot \cos(p_{3})+y\cdot \sin(p_{3})]\\ \;+\frac{\partial g(S_{p},\bar{P})}{\partial \tilde{y}}\cdot [-x\cdot \sin(p_{3})-y\cdot \cos(p_{3})]. \end{array}$$

When $\bar{P}$ is close to the exact solution,

(10) $$g(S_{p},\bar{P})\approx f(S_{p})$$

so that the first sum item of Eq. (8) can be ignored and we will get

(11) $$\begin{array}{l} \nabla \nabla C(\bar{P})\approx \frac{2}{\sum_{S_{p}\in f,{\tilde{S}}_{p}\in g}f^{2}(S_{p})}\cdot \\ \sum_{S_{p}\in f,{\tilde{S}}_{p}\in g}\frac{\partial g(S_{p},\bar{P})}{\partial p_{i}}\frac{\partial g(S_{p},\bar{P})}{\partial p_{j}}. \end{array}$$

## 3. Definitions and Measures of Registration

In order to quantify the reproducibility of the topography of the SRM bullets and cartridge cases, we use two parameters that compare pairs of 3D images or 2D profiles, the cross-correlation function maximum (*CCF_max_*) and a new parameter, the signature difference *D_s_* [9]. Given two casing signature images, denoted as *f* and *g* following the registration algorithm description in Sec. 2, we take *f* as reference and transform the position of *g* using three parameters, horizontal translation *T_x_*, vertical translation *T_y_*, and rotation angle *θ*. For a certain group of variants (*T_x_*, *T_y_*, *θ*), a corresponding image $g^{\prime }$ transformed from *g*, and the overlapping subarea of *f* and $g^{\prime }$, which are denoted as *f_s_* and ${g^{\prime }}_{s}$, are determined. In the image processing application, the normalized cross-correlation function will be calculated as

(12) $$CCF(f,g,T_{x},T_{y},\theta )=CCF(f_{s},{g^{\prime }}_{s})=\langle \frac{f_{s}}{\left\|f_{s}\right\|}\cdot \frac{{g^{\prime }}_{s}}{\left\|{g^{\prime }}_{s}\right\|}\rangle$$

where <⋅,⋅> is the inner product and ‖⋅‖ is the *L*^2^-norm.

The maximum value *CCF_max_* occurs when the two correlated surface topographies are registered at their best matched position. Although the *CCF_max_* can be used for casing signature comparison, it is not sensitive to *z*-scale differences. Assuming two compared signatures have the same shape but different amplitude scales, their *CCF_max_* is still 100 %. Therefore another parameter called the signature difference, *D_s_*, which is highly correlated with the *CCF_max_* but directly quantifies bullet profile signature difference, has also been proposed [9].

The parameter *D_s_* is calculated in the following way. After the registration algorithm is performed, a transformed image $g^{\prime }$ which is at the best position to match the reference image *f* is determined. At this position, the overlapping areas in image *f* and image $g^{\prime }$ are denoted as *f_s_*_,0_ and ${g^{\prime }}_{s,0}$, respectively. The signature difference *D_s_* is defined as a ratio of the area-based mean-square roughness *Sq*^2^ of the topography difference ${g^{\prime }}_{s,0}-f_{s,0}$ and the mean-square roughness of the reference topography *f*_s,0_. The parameter *D_s_* therefore casts the mean square difference as a fraction of the mean square roughness of the reference topography, so for good registration of similar surfaces the value of *D_s_* should be close to zero

(13) $$D_{s}=\frac{Sq^{2}({g^{\prime }}_{s,0}-f_{s,0})}{Sq^{2}(f_{s,0})}.$$

For a set *v* having *n* elements, the definition of the mean-square roughness *Sq*^2^ is

(14) $$Sq^{2}(v)=\frac{1}{n}\sum_{i=1}^{n}v_{i}{}^{2}.$$

Equation (13) and Eq. (3) have the same physical meaning and they can be shown to be equivalent to one another. More specifically, the parameter *D_s_* is calculated after the transform parameter **P** is solved and two images are aligned, whereas **P** is solved through minimizing *C*, a parameter directly equivalent to *D_s_*. As a result, our registration algorithm, which searches a set of optimum transformation parameters to minimize the function *C* in Eq. (3), ensures the consistency between the image registration and the calculation of *D_s_*. It avoids possible error caused by inconsistent criteria between registration and similarity measurement.

## 4. Experimental Data and Results

**Fig. 1:**
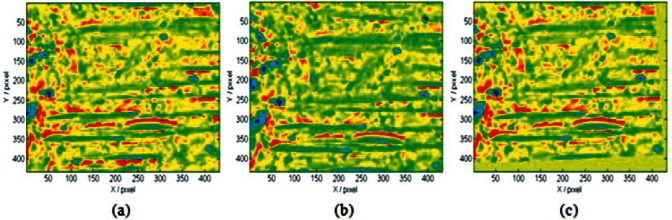
Images of comparable areas of breech face impressions on two NIST standard cartridge cases acquired by confocal microscopy after Gaussian filtering shown as (a) and (b). Fig. 1(c) is the transform of (b) using the algorithm proposed in Sec. 2 at the position of optimum registration, *CCF_max_* = 97.9 %.

The algorithm has been tested by registering three pairs of topographic images of standard cartridge cases to determine how similar they are. In all three cases very good correlation is obtained. An example is given as follows. Images shown in Fig. 1(a) and 1(b) are portions of topographic images of breech face impressions acquired from two different SRM cartridge cases by a commercial confocal microscope. They are filtered using a Gaussian filter [13] with 0.08 mm long-wavelength cutoff to remove the low frequency components of the original casing surface, i.e., the curvature and waviness. The filter process accentuates the individual characteristics impressed by the firearm. This system originally used an open source code to register different breech face or firing pin images before the similarity calculation [14]. However, during our recent test for breech face signature measurements, the software sometimes failed to register two correlated images at their best matched position. In this example, the *CCF_max_* calculated between two images after registration gave an extremely low value, *CCF_max_* = 5.5 %. With the image in Fig. 1(a) selected as the reference, the image in Fig. 1(b) does not show any positional transform to match the reference after the registration process, indicating that the open source registration process did not work for this case. This error does not happen when applying the new registration algorithm described in Sec. 2. By applying this algorithm, an optimal array of transformation parameters **P** is determined. Then the comparison image is transformed back to match the reference image using the array **P** as shown in Fig. 1(c). The resulting similarity metrics are *CCF_max_* = 97.9 % and *D_s_* = 4.2 %. Taking the center point of the image as coordinate origin, the calculated transformation parameters are *T_x_* = 16.3 pixels, *T_y_* = 32.9 pixels and *θ* = 2.9°.

All NIST standard cartridge cases are electroformed replicas from the same master casing provided by the ATF. High agreement and reproducibility between the casing signatures of different standard casings have been verified previously [15]. In this experiment, a 0.08 mm long-wavelength cutoff Gaussian filter has been adopted. This filter procedure emphasizes shorter surface spatial wavelengths than the 0.25 mm cutoff mainly used in our previous experiments and eliminates more components considered as low frequency waviness of the topography. With this procedure, the experimental results still show that the proposed registration algorithm is an effective method for establishing the similarity of NIST standard cartridge cases and is capable of registering surfaces with sub-pixel precision.

## 5. Discussion

The automatic algorithm proposed in this paper solves the 2D image registration problem for rigid body transformation consisting of *x*- and *y*-translation and *z*-rotation. Furthermore, for an affine [2] transformation of images with different magnification scales, in case, for example, two images were measured with different lateral magnifications, this algorithm can be further expanded by adding a scaling factor *l*. The first and second order derivative matrix for implementing the optimization can then be derived from the following equation:

$$\{\begin{array}{c} \tilde{x}=T_{x}+l\cdot (x\cdot \cos\theta -y\sin\theta )\\ \tilde{y}=T_{y}+l\cdot (x\cdot \sin\theta +y\cos\theta ) \end{array}.$$ (15)

## Footnotes

1 Certain commercial equipment, instruments, or materials are identified in this paper to foster understanding. Such identification does not imply recommendation or endorsement by the National Institute of Standards and Technology, nor does it imply that the materials or equipment identified are necessarily the best available for the purpose.
